# Analysis of anatomic location of burns inpatients in China from 2009 to 2018

**DOI:** 10.1186/s12889-024-18910-2

**Published:** 2024-07-05

**Authors:** Jie Yang, Jianchao Liu, Kui Ma, Huajuan Bai, Mingzi Ran, Guanglei Tian, Siming Yang, Xiaobing Fu

**Affiliations:** 1 Research Center for Tissue Repair and Regeneration affiliated to the Medical Innovation Research Department, PLA General Hospital and PLA Medical College; PLA Key Laboratory of Tissue Repair and Regenerative Medicine and Beijing Key Research Laboratory of Skin Injury, Repair and Regeneration; Research Unit of Trauma Care, Tissue Repair and Regeneration, Chinese Academy of Medical Sciences, 2019RU051, Beijing, 100048 PR China; 2 Department of Dermatology, The Fourth Medical Center, PLA General Hospital, Beijing, 100048 PR China; 3 Institution of Hospital Management, Chinese PLA General Hospital, Beijing, 100853 PR China; 4 Department of Anesthesiology, The Fourth Medical Center, PLA General Hospital, Beijing, 100048 PR China

**Keywords:** Burns, Anatomic locations of burns, China, Clinical characteristic

## Abstract

**Background:**

Burns cause serious physical and psychological harm to patients, placing a heavy burden on the global healthcare system. Our previous study detailed the epidemiological characteristics of burn injuries in Chinese inpatients from 2009 to 2018. Interestingly, the anatomic locations of burn injuries vary by gender, age, provinces, and outcomes among different causes. Therefore, this current study aims to analyze the characteristics of burn injuries in inpatients with various burn sites by collecting data in China from 2009 to 2018. This analysis will inform future healthcare system decisions and provide effective strategies.

**Methods:**

Burns inpatients from 196 hospitals across 31 provinces in China were included in the study, covering the period from 2009 to 2018. The data collected encompassed information on gender, age, etiology, regions, clinical outcomes, and anatomical locations of the injuries. Data analysis was conducted using Microsoft Excel 2007.

**Results:**

From 2009 to 2018, a total of 333,995 burns inpatients were recorded. The most vulnerable parts to burns were multiple burn sites (230,090, 68.89%). Women were more susceptible to lower limb burns (15,608, 14%), while men were more prone to eye injuries (8,387, 3.37%) and hand burns (6,119, 2.75%). The age group of 0–10 years was the most vulnerable to burns across all body areas, including internal organs. In China, individuals aged 20–50 years were at a higher risk of head and neck burns compared to other age groups. The Han population showed increased vulnerability to eye injuries (2.12 times higher than minorities), respiratory tract issues (2.09 times higher than minorities), and trunk burns (1.83 times higher than minorities), while being less susceptible to internal organ injuries (0.23 times fewer than minorities) and lower limb burns (0.78 times fewer than minorities). The southwest region had the highest proportion of burns inpatients with burns affecting single body parts, whereas the eastern area had the highest rates of respiratory tract burns (0.85%) and multiple burn sites (80.64%). Scalding was identified as the most common cause of burns, while flame burns (769, 55.81%) and chemical burns (438, 47.35%) were the main causes of respiratory tract and internal organ injuries, respectively.

**Conclusions:**

This study provides an initial description of characteristics of burns inpatients with various anatomic locations of burns in China over the past decade. Our findings will contribute to the most up-to-date clinical evidence database for healthcare planning and prevention initiatives in both China and other countries.

## Background

Burns represent a global public health problem, accounting for an estimated 180,000 deaths annually. They not only cause serious physical and psychological harm to patients but also place a heavy burden on the global healthcare system [1–4]. In developing countries, the vast majority (96%) of deaths are from fire-related burns, making burns one of the leading causes of disability-adjusted life-years (DALYs) [5]. Moreover, the ratio of pediatric deaths caused by burns is currently over 7 times higher in developing countries than in developed countries [4]. Although clinical treatments have improved over time such as fluid resuscitation, early enteral nutrition and aggressive surgery, the mortality rate for burns remains still high, especially in the developing world [6, 7]. In addition, various types of work, as well as local environments and cultures, can result in different types of burn injuries among patients [8–11]. As a result, regionalizing and proposing targeted burn prevention strategies according to the nature of work and local environments is a top priority for future social development worldwide.

Our previous study precisely described the epidemiological characteristics of burn inpatients in China from 2009 to 2018 [12]. Interestingly, burns occurring at different anatomic locations can lead to various outcomes for burn patients [13]. Different anatomic locations of burns exhibit variations in terms of gender, age, and etiology [14]. Additionally, in our country, different provinces may have varying susceptibilities to specific locations of burns due to the nature of local work, culture, and gross domestic product (GDP), which can also influence the etiology of burns [15–17]. Notably, there has been no systematic analysis of burn sites for burn prevention in China. Therefore, in this study, we recruited 196 hospitals of different sizes from 31 administrative and provincial regions across the country from 2009 to 2018, and analyzed the clinical characteristics of anatomic locations of burns.

## Methods

### Patient selection

The method of collection data was described in previous study with slight modification [12]. The present study has collected 333,995 inpatients from the hospitals across mainland China from 2009 to 2018 by non-probability sampling performing structured query language (SQL). Inpatient data were excluded if any primary factors were missing, including hospitalization and diagnosis information, as well as patient identity. Additionally, the following data were obtained from hospital electronic medical records: the etiology of burn injuries including flame burns, scald burns, electrical burns, chemical burns, and explosion burns. The etiology, diagnosis, and outcomes of inpatients were classified according to the International Classification of Diseases 10 (ICD-10).

### Statistical analysis

Our analysis was based on the hospital disease profiles of inpatients from over 100 representative cities, including provincial capitals and key regions, accounting for approximately 30% of all cities in China [12]. The data collected were presented as numbers and percentages and were primarily processed using Microsoft Excel 2007.

## Results

### Anatomic locations of burns and genders

**Fig. 1:**
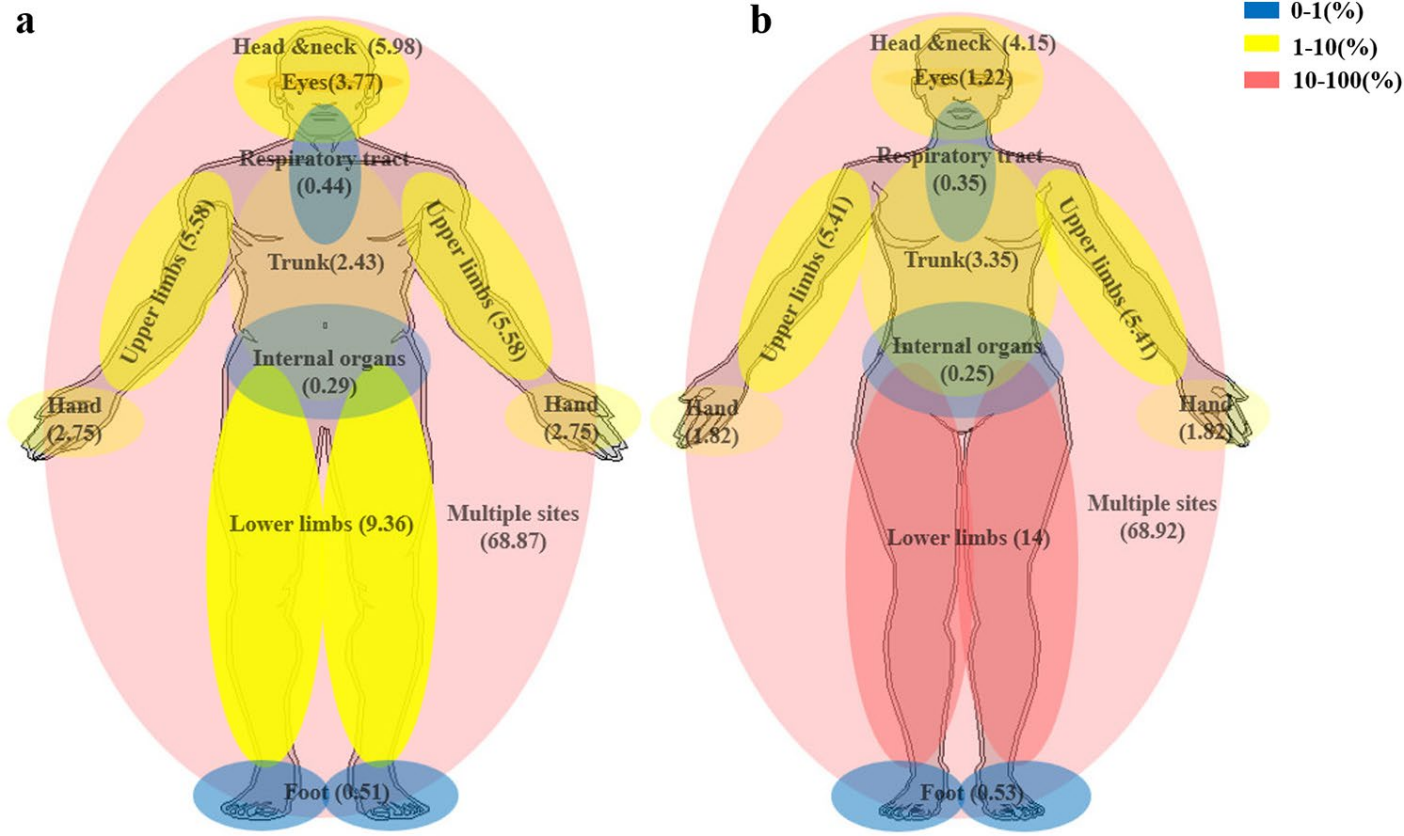
The changes of different anatomic locations of burns among genders from 2009–2018. **a**. Ratio of male inpatients affected by burns across different anatomic locations of burns. **b**. Ratio of female inpatients affected by burns across different anatomic locations of burns

Number of burns inpatients was 333,995, including 222,480 male(66.61%) and 111,515 female(33.39%). Multiple sites were the highest ratio of the different burns sites to total burns inpatients in both male(153,230, 68.87%) and female(76,860, 68.92%). The second highest ratio of burns in male inpatients were lower limbs (20,831, 9.36%), head and neck (13,297, 5.98%), and upper limbs (12,425, 5.58%). For female burn inpatients, the second highest ratio was lower limbs (15,608, 14%), followed by upper limbs (6,033, 5.41%), and head and neck (4,626, 4.15%). Internal organs had the lowest ratio for both male (647, 0.29%) and female (278, 0.25%) burn inpatients. Moreover, women were more vulnerable to lower limbs burns compared to men, while men were more vulnerable to eyes(8387, 3.37%) and hand(6119, 2.75%) compared to women’s eyes(1358, 1.22%) and hand(2035, 1.82%) (Fig. 1).

### Anatomic location of burns and age with etiology

**Fig. 2:**
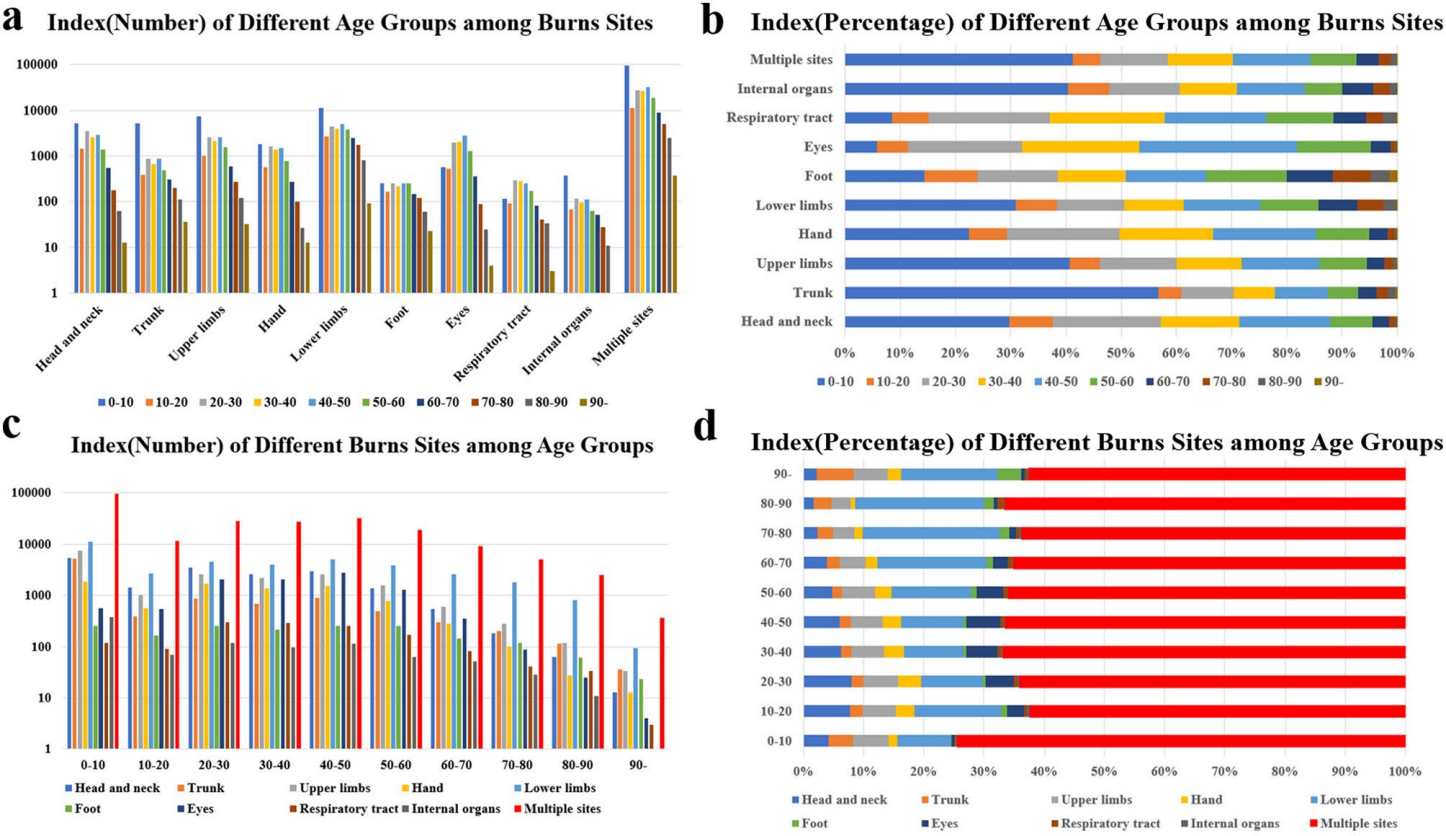
The changes between different anatomic locations of burns and age groups. **a**. Number of burns inpatients with different anatomic locations of burns changes among age groups. **b**. The percentage of burns inpatients with different anatomic locations of burns changes among age groups. **c**. Number of burns inpatients in different age groups changes among anatomic locations of burns. **d**. The percentage of burns inpatients in different age groups changes among anatomic locations of burns

The most common anatomic locations of burns in children aged 0–10 years were head and neck (29.7%), trunk (40.66%), upper limbs (56.67%), hand (22.46%), lower limbs (30.95%), internal organs (40.43%), and multiple sites (41.21%). This was followed by the age groups 20–30 years (19.48%, 9.52%, 13.91%, 20.36%, 12.25%, 12.65%, and 12.23% respectively) and 40–50 years (16.46%, 9.63%, 14.14%, 18.52%, 13.8%, 12.32%, and 14.15% respectively) in the population. Furthermore, burns in special locations such as eyes (20.8%, 21.25%, and 28.57% respectively) and the respiratory tract (21.84%, 20.9%, and 18.29% respectively) were more common in the age groups of 20–30, 30–40, and 40–50, indicating that individuals of working age are more likely to experience burns in these areas (Fig. 2a, b).

Diagnosing multiple burn sites was most common across all age groups and showed a declining trend with age. The second most common location for burns was the lower limbs, which demonstrated an increasing trend with age. Burns occurring on the head and neck, eyes, and hands were more prevalent among individuals of working age, including those in the age groups of 20–30 (7.96%, 4.59%, and 3.78% respectively), 30–40 (6.31%, 5.13%, and 3.42% respectively), 40–50 (6.03%, 5.69%, and 3.08% respectively), and 50–60 (4.75%, 4.48%, and 2.72% respectively). Interestingly, trunk burns were commonly observed in children aged 0–10 (4.07%) and elderly individuals aged 90 and above (6.13%), indicating injuries among individuals with limited mobility (Fig. 2c, d).

**Fig. 3:**
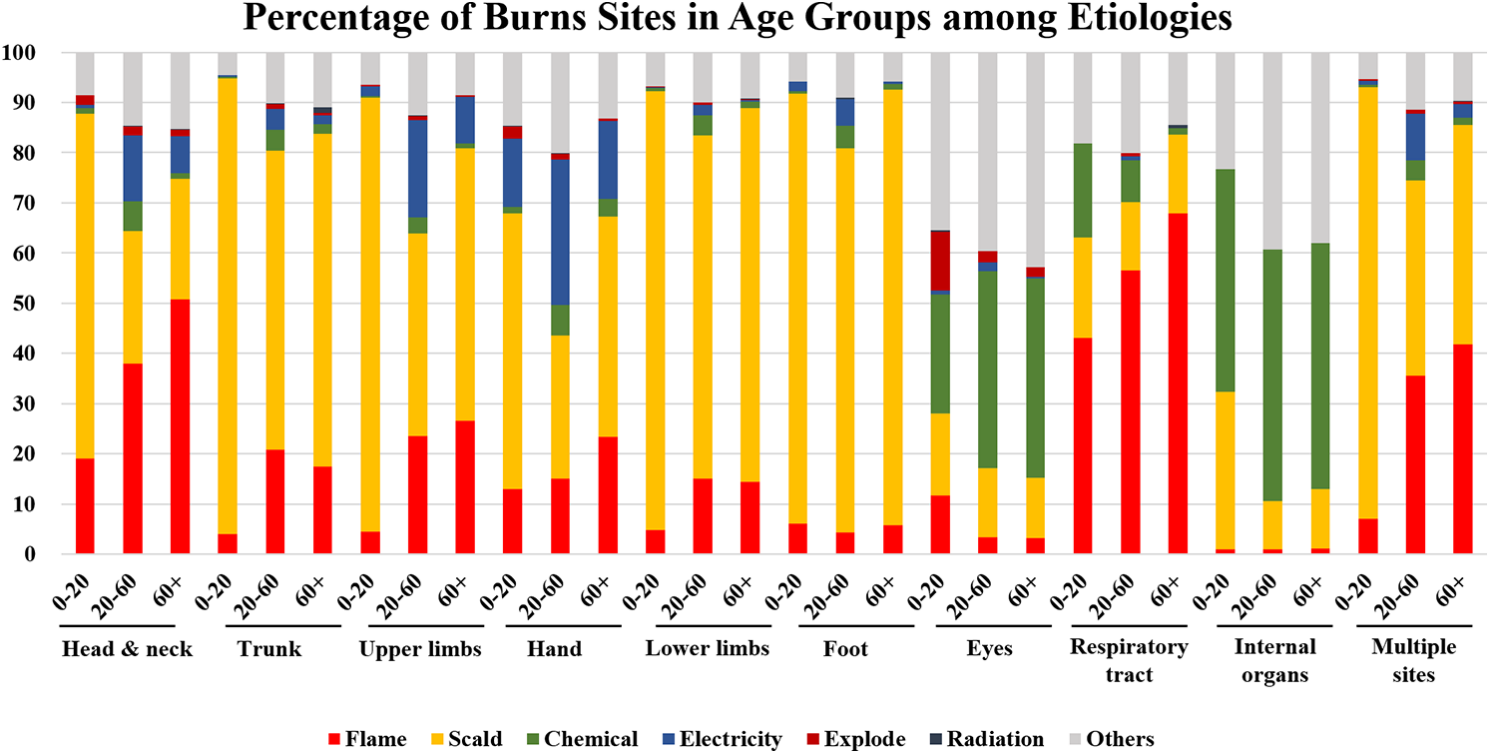
The percentage of anatomic locations of burns in age groups changes among etiologies. The age groups were divided according to underage, adult and elderly and the extent of body parts among different age groups vulnerable to burns was explicitly showed in the figure

To analyze burns sites of different etiologies across all age groups, we categorized the 10 age groups into 3 categories: 0–20, 20–60, and 60 + age groups representing minors, adults, and the elderly. Scalding was the most common cause of burns among all age groups and at various burn locations, followed by flame, electricity, and chemical burns. The 0–20 age group showed a higher susceptibility to scalding compared to other age groups. Flame burns were more prevalent on the upper body, particularly in the respiratory tract, with an increasing trend in percentage with age. Electricity burns were frequently observed on the upper limbs and hands across age groups, with the highest percentage occurring in the 20–60 age group (19.32% and 29.03%). Chemical burns were a common cause of eye injuries (37.48%) and internal organ damage (47.35%). Interestingly, chemical burns were also a common cause of respiratory tract injuries in the 0–20 age group (Fig. 3).

### Anatomic location of burns and ethnic group

**Fig. 4:**
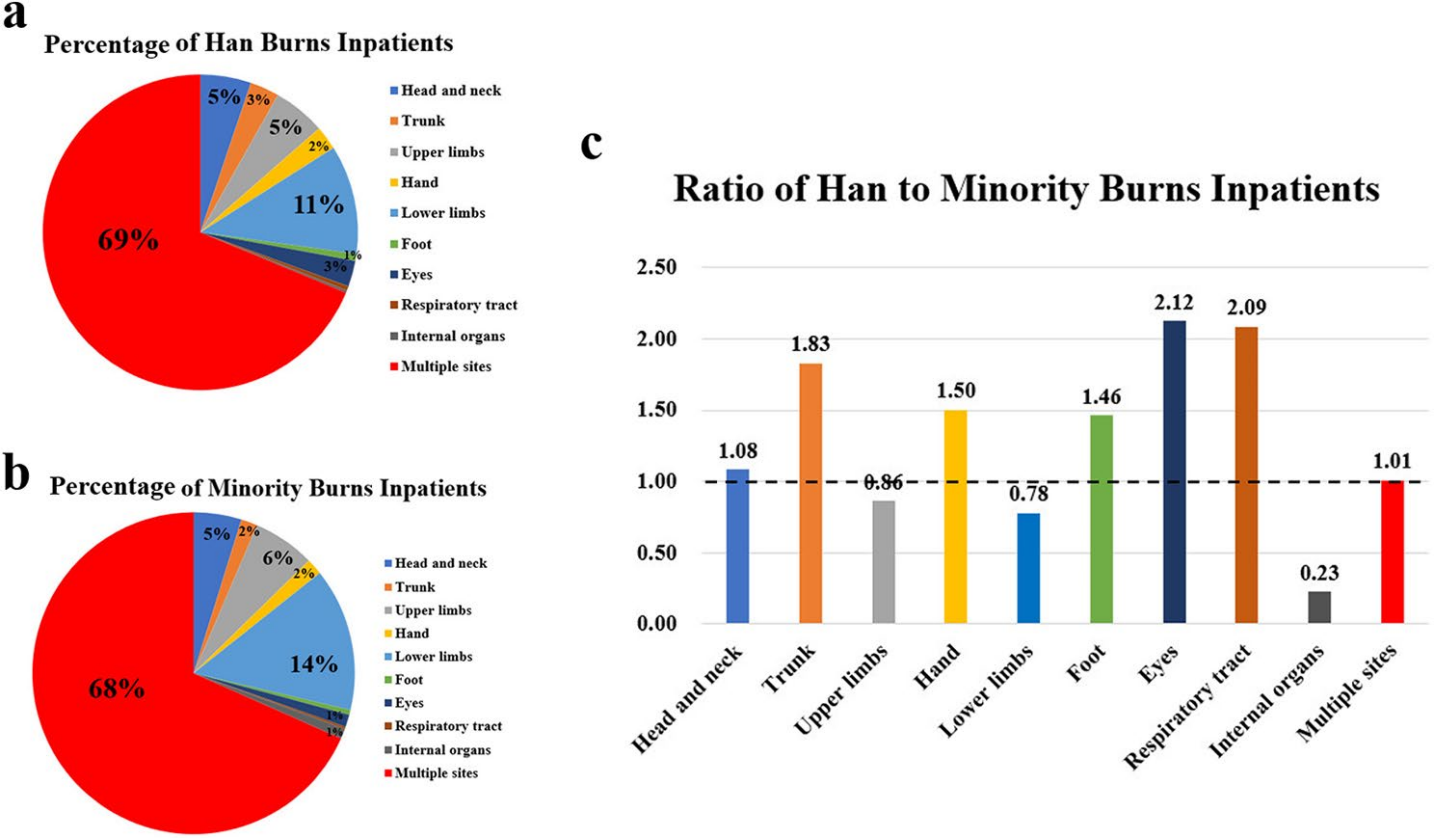
The changes between different burns sites and ethic group. **a**. Percentage of burns inpatients with different burns sites accounts for Han. **b**. Percentage of burns inpatients with different burns sites accounts for Minority. **c**. Ratio of burns inpatients with different burns sites changes in Han to Minority

Han is the largest ethnic group in China, which accounted for 91.11% population of China. Due to the smaller population of other ethnic minorities in China, they are often discussed collectively. The ethnic minorities include 55 groups other than the Han ethnicity. Multiple burn sites had the highest percentage of burns in patients among Han and minority groups, accounting for 68.75% and 68.35%, respectively. This was followed by lower limbs (11.17% and 14.37%) (Fig. 4a, b). Additionally, Han individuals were more vulnerable to eye burns (Han to minority ratio: 2.12), respiratory tract burns (Han to minority ratio: 2.09), and trunk burns (Han to minority ratio: 1.83), while minorities were more susceptible to internal organ injuries (Han to minority ratio: 0.23) and lower limb injuries (Han to minority ratio: 0.78) (Fig. 4c).

### Anatomic location of burns and provinces in China

Next, we analyzed the incidence of burn locations among provinces in China. The most common location for burns was multiple sites, which were especially prevalent in Xizang (87.87%), followed by Henan (87.32%) and Fujian (85.02%). Burns in locations other than the respiratory tract frequently occurred in Chongqing. The highest ratio of burns affecting the respiratory tract was in Anhui (2.3%), followed by Zhejiang (2.06%) and Tianjin (1.2%) (Table 1).

**Fig. 5:**
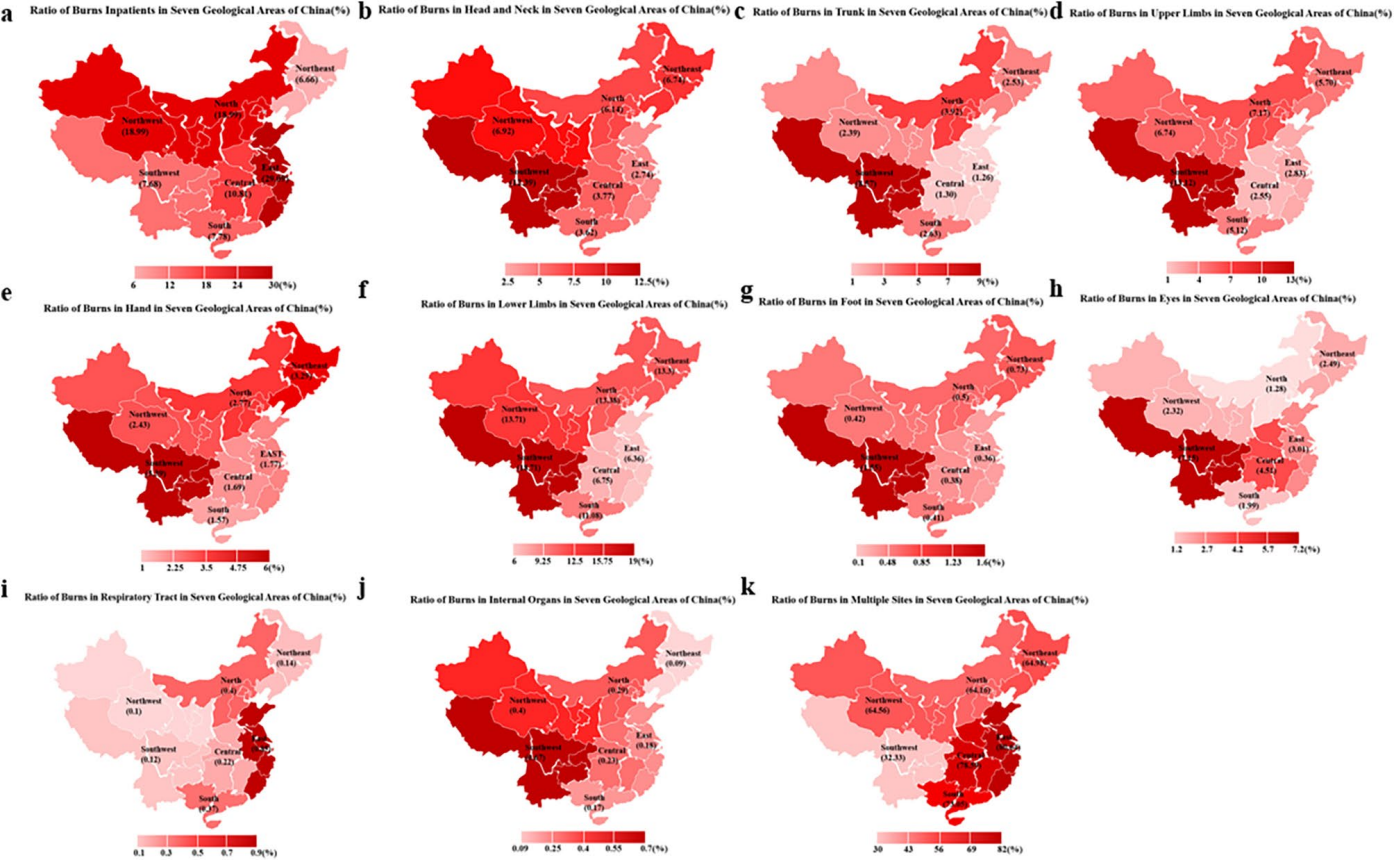
Ratio of different burns sites in seven areas of China. **a**. Ratio of burns inpatients in seven areas of China. **b**. Ratio of head and neck in total burns sites shows in seven areas of China. **c**. Ratio of trunk in total burns sites shows in seven areas of China. **d**. Ratio of upper limbs in total burns sites shows in seven areas of China. **e**. Ratio of hand in total burns sites shows in seven areas of China. **f**. Ratio of lower limbs in total burns sites shows in seven areas of China. **g**. Ratio of foot in total burns sites shows in seven areas of China. **h**. Ratio of eyes in total burns sites shows in seven areas of China. **i**. Ratio of respiratory tract in total burns sites shows in seven areas of China. **j**. Ratio of internal organs in total burns sites shows in seven areas of China. **k**. Ratio of multiple burns sites in total burns sites shows in seven areas of China

To further analyze the trends in different provinces of China, we divided all provinces in our study into seven areas based on geographical distribution. Our data revealed that the eastern area (29.09%) had the highest ratio of burn patients in China, followed by the northern (18.99%) and northwestern areas (18.99%). The southwestern region had the highest ratio of burn patients with burns occurring in single body parts, including the head and neck (12.39%), trunk (8.57%), upper limbs (13.12%), hands (5.39%), lower limbs (18.71%), feet (1.55%), eyes (7.15%), and internal organs (0.67%). In contrast, the eastern area had the highest ratio of burns affecting the respiratory tract (0.85%) and multiple burn sites (80.64%) (Fig. 5).

**Table 1:** Number (Ratio) of anatomic location of burns injuries occurred among provinces in China

Anatomic location of burn injuries	Head and neck	Trunk	Upper limbs	Hand	Lower limbs	Foot	Eyes	Respiratory tract	Internal organs	Multiple sites	Total
Provinces, n(%)											
Qinghai	259(11.32)	54(2.36)	62(2.71)	102(4.46)	128(5.59)	0	57(2.49)	1(0.04)	2(0.09)	1623(70.93)	2288
Jiangsu	704(3.95)	305(1.71)	570(3.2)	322(1.81)	1180(6.63)	47(0.26)	412(2.31)	38(0.21)	37(0.21)	14,195(79.7)	17,810
Hunan	226(4.04)	45(0.8)	206(3.68)	94(1.68)	364(6.51)	32(0.57)	229(4.09)	7(0.13)	7(0.13)	4384(78.37)	5594
Fujian	474(1.15)	347(0.84)	1068(2.59)	563(1.37)	2064(5.01)	45(0.11)	1340(3.25)	188(0.46)	84(0.2)	35,041(85.02)	41,214
Yunnan	485(9.38)	178(3.44)	325(6.29)	228(4.41)	764(14.78)	46(0.89)	638(12.34)	11(0.21)	13(0.25)	2482(48.01)	5170
Guizhou	7(9.33)	4(5.33)	4(5.33)	7(9.33)	10(13.33)	0	30(40)	0	0	13(17.33)	75
Zhejiang	1021(3.95)	337(1.3)	643(2.49)	356(1.38)	1705(6.6)	13(0.05)	325(1.26)	532(2.06)	11(0.04)	20,908(80.88)	25,851
Gansu	637(7.79)	155(1.9)	540(6.6)	187(2.29)	945(11.56)	9(0.11)	252(3.08)	5(0.06)	9(0.11)	5439(66.51)	8178
Beijing	891(4.21)	647(3.06)	1778(8.4)	622(2.94)	3949(18.67)	179(0.85)	346(1.64)	146(0.69)	129(0.61)	12,469(58.94)	21,156
Guangdong	263(3.6)	255(3.49)	283(3.87)	166(2.27)	489(6.69)	26(0.36)	152(2.08)	29(0.4)	15(0.21)	5635(77.05)	7313
Shaanxi	1597(15.33)	700(6.72)	1058(10.15)	539(5.17)	1174(11.27)	39(0.37)	310(2.98)	23(0.22)	58(0.56)	4918(47.22)	10,416
Chongqing	2196(17.69)	1939(15.62)	2597(20.92)	901(7.26)	3186(25.67)	296(2.38)	802(6.46)	14(0.11)	122(0.98)	362(2.92)	12,415
Xinjiang	1882(4.46)	604(1.43)	2612(6.18)	704(1.67)	6440(15.25)	214(0.51)	725(1.72)	37(0.09)	179(0.42)	28,835(68.28)	42,232
Ningxia	12(3.93)	1(0.33)	5(1.64)	11(3.61)	8(2.62)	3(0.98)	130(42.62)	0	4(1.3)	131(42.95)	305
Henan	492(2.06)	170(0.71)	263(1.1)	234(0.98)	711(2.98)	89(0.37)	960(4.02)	48(0.2)	56(0.23)	20,834(87.32)	23,857
Hebei	1712(8.81)	1176(6.06)	1148(5.91)	659(3.93)	1971(10.15)	113(0.58)	356(1.83)	60(0.31)	31(0.16)	12,195(62.79)	19,421
Tianjin	171(17.1)	98(9.8)	113(11.3)	59(5.9)	246(24.6)	6(0.6)	10(1)	12(1.2)	6(0.6)	279(27.9)	1000
Shanxi	308(4.77)	90(1.39)	266(4.12)	166(2.57)	375(5.81)	16(0.25)	33(0.51)	20(0.31)	13(0.2)	5166(80.01)	6453
Xizang	90(4.46)	8(0.4)	17(0.84)	29(1.44)	38(1.88)	1(0.05)	53(2.62)	1(0.05)	8(0.4)	1775(87.87)	2020
Jiangxi	152(9.28)	30(1.83)	106(6.47)	59(3.6)	230(14.04)	6(0.37)	183(11.17)	18(1.1)	8(0.49)	846(51.64)	1638
Shanghai	63(1.53)	74(1.8)	78(1.89)	93(2.26)	308(7.48)	19(0.46)	42(1.02)	27(0.66)	20(0.49)	3395(82.42)	4119
Hainan	516(4.27)	377(3.12)	817(6.76)	158(1.31)	1864(15.42)	42(0.35)	145(1.20)	20(0.17)	13(0.11)	8137(67.31)	12,089
Hubei	493(9.8)	226(4.49)	346(6.88)	224(4.45)	1132(22.5)	12(0.24)	256(5.09)	6(0.12)	13(0.26)	2324(46.18)	5032
Jilin	397(6.4)	62(1)	241(3.89)	206(3.32)	452(7.29)	89(1.44)	126(2.03)	5(0.08)	8(0.13)	4613(74.42)	6199
Sichuan	402(6.71)	71(1.19)	425(7.1)	219(3.66)	805(13.45)	54(0.90)	312(5.21)	4(0.07)	30(0.5)	3665(61.22)	5987
Liaoning	712(7.12)	287(2.87)	560(5.6)	358(3.58)	1251(12.5)	62(0.62)	382(3.82)	19(0.19)	7(0.07)	6368(63.64)	10,006
Inner Mongolia	810(5.26)	476(3.09)	1242(8.07)	251(1.63)	1947(12.65)	2(0.01)	67(0.44)	13(0.08)	3(0.02)	10,578(68.74)	15,389
Shandong	342(4.92)	155(2.23)	362(5.2)	346(4.97)	905(13.01)	221(3.18)	672(9.66)	12(0.17)	16(0.23)	3926(56.43)	6957
Anhui	60(4.94)	11(0.91)	25(2.06)	39(3.21)	20(1.65)	6(0.49)	134(11.03)	28(2.3)	4(0.33)	888(73.09)	1215
Heilongjiang	389(6.46)	214(3.55)	467(7.75)	168(2.79)	1253(20.79)	12(0.2)	46(0.76)	8(0.13)	4(0.07)	3465(57.5)	6026
Guangxi	160(2.43)	50(0.76)	231(3.52)	84(1.28)	525(7.99)	38(0.58)	220(3.35)	46(0.7)	15(0.23)	5201(79.16)	6570

### Anatomic location of burns and etiology with outcomes

To optimize the prevention of burns, we conducted a detailed analysis of the correlation between burn sites, causes, and outcomes. Our findings revealed that, with the exception of internal organs and the respiratory tract, the majority of burn locations were associated with a high risk of mortality following explosions. Specifically, internal organs and the respiratory tract accounted for 0.23% and 2.4% of burn-related fatalities after chemicals, respectively. We further examined the distribution of outcomes for the same causes across different burn sites. It was observed that burns affecting multiple sites were most prevalent across various causes. In cases of flame, scald, and chemical burns, the highest mortality rates were typically observed in the respiratory tract, with percentages of 1.82%, 1.95%, and 2.4%, respectively. Conversely, in electrical and explosion-related burns, the respiratory tract exhibited the highest rate of recovery at 100%, while the trunk had the highest mortality rates, at 1.99% and 5.88% for electricity and explosions, respectively (Table 2).

**Table 2:** Number (Ratio) of outcomes in anatomic location of burn injuries occurred among etiologies

Anatomic location of burn injuries	Outcomes	Flame	Scald	Chemical	Electricity	Explode	Radiation	Others	Total
	n(%)								
Head and neck	Improved	1499(26.59)	2561(33.87)	200(28.74)	454(30.82)	85(25.53)	6(66.67)	704(31.8)	5509
	Cured	4090(72.56)	4948(65.44)	492(70.69)	1014(68.84)	238(71.47)	3(33.33)	1491(67.34)	12,276
	Ineffective	1(0.02)	0	0	0	0	0	2(0.09)	3
	Death	9(0.16)	1(0.01)	0	0	2(0.6)	0	1(0.05)	13
	Untreated	10(0.18)	16(0.21)	2(0.29)	2(0.14)	1(0.3)	0	6(0.27)	37
	Others	28(0.5)	35(0.46)	2(0.29)	3(0.2)	7(2.1)	0	10(0.45)	85
	Total	5637(31.45)	7561(42.19)	696(3.88)	1473(8.22)	333(1.86)	9(0.05)	2214(12.35)	17,923
Trunk	Improved	400(42.33)	3419(47.25)	72(48.98)	38(25.17)	5(14.71)	5(38.46)	220(35.48)	4159
	Cured	522(55.24)	3760(51.96)	72(48.98)	110(72.85)	27(79.41)	8(61.54)	396(63.87)	4895
	Ineffective	2(0.21)	5(0.07)	0	0	0	0	0	7
	Death	9(0.95)	9(0.12)	2(1.36)	3(1.99)	2(5.88)	0	0	25
	Untreated	2(0.21)	14(0.19)	0	0	0	0	3(0.48)	19
	Others	10(1.06)	29(0.4)	1(0.68)	0	0	0	1(0.16)	41
	Total	945(10.33)	7236(79.12)	147(1.61)	151(1.65)	34(0.37)	13(0.14)	620(6.78)	9146
Upper limbs	Improved	841(30.58)	5136(44.58)	130(39.27)	559(28.29)	30(28.04)	3(50)	650(36.79)	7349
	Cured	1885(68.55)	6324(54.89)	201(60.73)	1407(71.2)	74(69.16)	3(50)	1104(62.48)	10,998
	Ineffective	1(0.04)	1(0.01)	0	0	1(0.93)	0	1(0.06)	4
	Death	5(0.18)	2(0.02)	0	1(0.05)	0	0	0	8
	Untreated	1(0.04)	23(0.2)	0	3(0.15)	1(0.93)	0	2(0.11)	30
	Others	17(0.62)	35(0.3)	0	6(0.3)	1(0.93)	0	10(0.57)	69
	Total	2750(14.9)	11,521(62.42)	331(1.79)	1976(10.71)	107(0.58)	6(0.03)	1767(9.57)	18,458
Hand	Improved	411(33.97)	996(32.97)	156(42.05)	696(35.84)	43(35.83)	5(41.67)	549(37.14)	2856
	Cured	787(65.04)	1991(65.91)	212(57.14)	1228(63.23)	77(64.17)	7(58.33)	909(61.5)	5211
	Ineffective	0	1(0.03)	0	0	0	0	0	1
	Death	0	0	0	0	0	0	0	0
	Untreated	1(0.08)	16(0.53)	2(0.54)	7(0.36)	0	0	10(0.68)	36
	Others	11(0.91)	17(0.56)	1(0.27)	11(0.57)	0	0	10(0.68)	50
	Total	1210(14.84)	3021(37.05)	371(4.55)	1942(23.82)	120(1.47)	12(0.15)	1478(18.13)	8154
Lower limbs	Improved	1366(34.05)	11,380(40.77)	218(44.17)	133(32.13)	33(37.08)	4(33.33)	1126(35.62)	14,260
	Cured	2516(62.71)	16,365(58.63)	617(25.95)	277(66.91)	51(57.3)	7(58.33)	1994(63.08)	21,827
	Ineffective	1(0.02)	5(0.02)	1(73.45)	0	0	1(8.33)	3(0.09)	11
	Death	40(1)	20(0.07)	2(0.12)	1(0.24)	2(2.25)	0	2(0.06)	67
	Untreated	49(1.22)	61(0.22)	1(0.24)	0	0	0	26(0.82)	137
	Others	40(1)	80(0.29)	1(0.12)	3(0.72)	3(3.37)	0	10(0.32)	137
	Total	4012(11.01)	27,911(76.6)	840(2.31)	414(1.14)	89(0.24)	12(0.03)	3161(8.67)	36,439
Foot	Improved	31(35.63)	634(45.16)	23(46)	18(29.51)	0	1(33.33)	50(37.88)	757
	Cured	56(64.37)	763(54.34)	27(54)	43(70.49)	0	2(66.67)	80(60.61)	971
	Ineffective	0	0	0	0	0	0	0	0
	Death	0	2(0.14)	0	0	0	0	0	2
	Untreated	0	2(0.14)	0	0	0	0	2(1.52)	4
	Others	0	3(0.2)	0	0	0	0	0	3
	Total	87(5.01)	1404(80.83)	50(2.88)	61(3.51)	0	3(0.17)	132(7.6)	1737
Eyes	Improved	105(24.94)	474(34.67)	1347(36.88)	33(21.44)	124(39.62)	4(30.77)	1389(36.31)	3476
	Cured	312(74.11)	883(64.59)	2242(61.39)	121(78.57)	187(59.74)	9(69.23)	2411(63.03)	6165
	Ineffective	0	1(0.07)	2(0.05)	0	0	0	3(0.08)	6
	Death	0	0	0	0	0	0	0	0
	Untreated	2(0.48)	2(0.15)	41(1.12)	0	0	0	9(0.24)	54
	Others	2(0.48)	7(0.51)	20(0.55)	0	2(0.64)	0	13(0.34)	44
	Total	421(4.32)	1367(14.03)	3652(37.48)	154(1.58)	313(3.21)	13(0.13)	3825(39.25)	9745
Respiratory tract	Improved	187(24.32)	68(33.17)	63(50.4)	0	0	1(100)	108(40.91)	427
	Cured	550(71.52)	131(63.9)	59(47.2)	8(100)	6(100)	0	150(56.82)	904
	Ineffective	2(0.26)	1(0.49)	0	0	0	0	1(0.38)	4
	Death	14(1.82)	4(1.95)	3(2.4)	0	0	0	1(0.38)	22
	Untreated	5(0.65)	0	0	0	0	0	1(0.38)	6
	Others	11(1.43)	1(0.49)	0	0	0	0	3(1.14)	15
	Total	769(55.81)	205(14.88)	125(9.07)	8(0.58)	6(0.44)	1(0.07)	264(19.16)	1378
Internal organs	Improved	3(33.33)	50(26.74)	329(75.11)	0	0	0	207(71.13)	589
	Cured	6(66.67)	131(70.05)	87(19.86)	0	0	0	72(24.74)	296
	Ineffective	0	0	3(0.68)	0	0	0	1(0.34)	4
	Death	0	0	1(0.23)	0	0	0	4(1.37)	5
	Untreated	0	1(0.53)	8(1.83)	0	0	0	1(0.34)	10
	Others	0	5(2.67)	10(2.28)	0	0	0	6(2.06)	21
	Total	9(0.97)	187(20.22)	438(47.35)	0	0	0	291(31.46)	925
Multiple sites	Improved	16,696(31.82)	54,099(38.47)	1397(29.1)	2859(25.4)	397(28.52)	12(16.9)	7487(38.45)	82,947
	Cured	34,471(65.69)	85,475(60.78)	3351(69.8)	8306(73.8)	929(66.74)	59(83.1)	11,557(59.35)	144,148
	Ineffective	158(0.3)	67(0.05)	5(0.1)	5(0.04)	2(0.14)	0	13(0.07)	250
	Death	727(1.39)	241(0.17)	24(0.5)	24(0.21)	54(3.88)	0	164(0.84)	1234
	Untreated	129(0.25)	333(0.24)	9(0.19)	28(0.25)	2(0.14)	0	86(0.44)	587
	Others	291(0.55)	413(0.29)	15(0.31)	33(0.29)	8(0.57)	0	164(0.84)	924
	Total	52,472(22.8)	140,628(61.12)	4801(2.09)	11,255(4.89)	1392(0.6)	71(0.03)	19,471(8.46)	230,090

## Discussion

In our database, the multiple locations of burns had the highest ratio of burns among burn inpatients. This may be due to the fact that most burn cases among inpatients are caused by severe burns, which refer to extensive burns that affect multiple body parts [18]. In terms of gender distribution, men were more susceptible to burns on their eyes and hands, whereas women were more prone to burns on their lower limbs, often related to their respective occupations. This gender discrepancy in burn locations could be attributed to the differing occupational hazards between men and women, suggesting potential new strategies for burn prevention [7, 13, 19, 20].

Among the different age groups in our research, it was found that children in the 0–10 age group were the most vulnerable to burns all over their body, including internal organs. This indicates a lack of effective parental supervision and potential exposure to corrosive substances [21]. Moreover, rehabilitation of children with burns may require more special care [22, 23]. Burns occurring on the eyes and respiratory tract were more common among individuals aged 20–50, who are typically within the working age range. These types of burns are associated with a higher risk of disability and death, which can significantly impact future work and life. It is crucial for our country to prioritize the prevention of these injuries [24–26]. Interestingly, trunk burns were frequently observed in children aged 0–10 and elderly individuals aged 90 and above. This highlights the need for increased attention to preventing trunk burns in individuals with limited mobility. Additionally, individuals aged 60 and above were found to be more susceptible to flame burns in the respiratory tract, possibly due to their limited mobility. Our data also revealed that electricity burns were commonly seen on the upper limbs and hands of the working-age population, while chemical burns were a frequent cause of respiratory tract injuries among children and with high death rate. These findings underscore the importance of the government continuing to enhance safety measures for workers and to protect children and adolescents from hazardous locations. Education on dangerous chemicals should also be promoted to prevent such incidents.

Between the Han and minorities, minorities were more vulnerable to internal organ issues compared to the Han. This result is consistent with our previous study, which indicated that in Guizhou, minorities were more likely to suffer from chemical burns and there was a higher proportion of minority populations [27]. Additionally, the Han population was found to be more susceptible to eye and respiratory tract problems, possibly due to the nature of their occupations. These issues should be addressed by the government to prevent further health risks.

Different provinces exhibit varying patterns of burn injuries, with Chongqing showing a higher incidence of burns in different locations but a lower incidence of burns in multiple sites. This phenomenon may be attributed to the culinary culture in Chongqing and suggests that the local government has implemented effective fire prevention measures [28]. In contrast, individuals in Anhui and Zhejiang are more prone to respiratory tract burns, possibly due to the predominant industries in these regions such as new energy, new materials, and new generation information technology. This highlights the importance of establishing comprehensive fire protection policies by the respective local governments. Meanwhile, despite having a higher proportion of minority populations, people in Ningxia and Yunnan are more likely to experience eye burns compared to the Han population. This raises an intriguing hypothesis that Han individuals residing alongside local minorities may have a higher risk of eye burns. Additionally, Ningxia, Chongqing, and Beijing have the highest ratio of internal organ burns, potentially influenced by dietary habits in these areas. Further research is needed to explore this correlation in more depth.

In a study spanning seven regions of China based on geographical distribution, it was observed that the eastern region had a higher number of burn patients presenting with burns in multiple sites and respiratory tract injuries compared to other regions, particularly the southwest. The eastern region of our country is characterized by greater economic development, increased industrialization, and serves as a major trading port handling more cargo than other areas. This, combined with our findings, suggests that more severe burn cases occurred in the eastern region of China between 2009 and 2018 compared to other regions. This phenomenon underscores the need for enhancing fire safety and prevention measures in the eastern region [29].

In the section on anatomic location of burns and etiology with outcomes, burns of undetermined origin had a relatively high proportion among all causes. This could be attributed to the lack of clear or documented causes of burns in the diagnosis, as noted by medical professionals in various hospitals resulting in no definitive diagnosis following ICD-10 coding. The findings underscore the critical need to enhance the accuracy of doctors’ diagnoses for burn patients, particularly in accordance with ICD-10 guidelines. Scalding was identified as the most common cause of burns, consistent with our previous research and other studies [30, 31]. However, burns affecting the respiratory tract and internal organs were predominantly caused by flames and chemicals, respectively. Electric burns commonly affected the hands and upper limbs, with hand burns occurring nearly twice as frequently as burns to the upper limbs. This suggests that while protective measures against electric burns have improved, additional preventative strategies for hand injuries, such as enhancing the insulation of workwear, are warranted. Burns from explosions were more likely to affect the head and neck followed by the hands, indicating that although the government has implemented more comprehensive explosion prevention policies, there is still room for improvement based on our data analysis.

Furthermore, fatalities resulting from burns were predominantly associated with explosions. In comparison to the mortality rate of explosion-related injuries in US military personnel deployed overseas (1.4–1.5%) between 2005 and 2006 [32], our data showed higher death rates for burns affecting the trunk (5.88%), lower limbs (2.25%), and multiple sites (3.88%), while lower rates were observed for other types of burns. This suggests that despite advancements in clinical treatment, there is still a lack of effective interventions for injuries to the trunk, lower limbs, and multiple sites caused by explosions. Additionally, deaths related to burns affecting internal organs and the respiratory tract were most commonly attributed to chemical exposure, highlighting the need for government policies aimed at reducing the toxicity and flammability of surrounding materials. Our findings also revealed that among burns caused by flames, scalds, and chemicals – which represent the primary etiologies of burn incidents – the respiratory tract was identified as the main site leading to fatal outcomes, consistent with previous discussions. Moreover, for burns caused by electricity and explosions, which primarily result from occupational hazards, fatalities were most frequently associated with injuries to the trunk. This underscores the importance of enhancing protection measures when individuals are exposed to explosion or electricity in their work or living environments, and emphasizes the necessity of developing more effective treatment strategies for trunk injuries.

## Conclusion

In light of the national focus on burn injuries in our country, it is crucial to closely monitor changes in the anatomical locations of burn injuries across different demographics such as gender, age groups, and geographical regions. By doing so, we can develop more comprehensive strategies for burn prevention and treatment. Despite significant advancements in both burn prevention and treatment measures, the high number of burn patients indicates that there is still a gap between the standards of care in developed countries and our own. Therefore, it is imperative for our government to take proactive steps to enhance health promotion, child supervision, labor protection, and other relevant initiatives to reduce the incidence of burn-related hospitalizations. Establishing a national database on burns in China is essential for effectively formulating policies for burn prevention and treatment. This database would enable us to gather accurate data, identify trends, and tailor interventions to address specific needs within different populations. By leveraging this information, we can work towards narrowing the gap in burn care between our country and more developed nations.

## Data Availability

All data generated or analysed during this study are included in this published article.

## Abbreviations

DALYs: Disability-adjusted life-years
GDP: Gross domestic product
SQL: Structured query language
ICD-10: International classification of diseases-10

## References

[1] Smolle C, Cambiaso-Daniel J, Forbes A, Wurzer P, Hundeshagen G, Branski L, Huss F, Kamolz L (2017). Recent trends in burn epidemiology worldwide: a systematic review. Burns: J Int Soc Burn Injuries.

[2] Anlatici R, Ozerdem OR, Dalay C, Kesiktaş E, Acartürk S, Seydaoğlu G (2002). A retrospective analysis of 1083 Turkish patients with serious burns. Part 2: burn care, survival and mortality. Burns: J Int Soc Burn Injuries.

[3] Ajami S, Lamoochi P (2014). Comparative study on National burn Registry in America, England, Australia and Iran. J Educ Health Promotion.

[4] WHO Media Center Fact Sheets. Burns [Internet] 2018. http://wwwwhoint/mediacentre/factsheets/fs365/en/.

[5] Forbinake N, Ohandza C, Fai K, Agbor V, Asonglefac B, Aroke D, Beyiha G (2020). Mortality analysis of burns in a developing country: a CAMEROONIAN experience. BMC Public Health.

[6] Lam NN, Hung NT, Duc NM, Luong NV (2021). Epidemiology and risk factors for death of Pediatric Burns in a developing country. An experience from the National burn Hospital. Annals Burns fire Disasters.

[7] Global regional. and National age-sex specific all-cause and cause-specific mortality for 240 causes of death, 1990–2013: a systematic analysis for the global burden of Disease Study 2013. Lancet (London, England) 2015, 385(9963):117–71. 10.1016/S0140-6736(14)61682-2 PMC4340604 25530442

[8] Gurbuz K, Demir M. Comparison of work- and non-work-related lower extremity burn injuries: a retrospective analysis. Journal of burn care & research: official publication of the American Burn Association 2022. 10.1093/jbcr/irac034 35290468

[9] Schneider J, Shie V, Espinoza L, Shapiro G, Lee A, Acton A, Marino M, Jette A, Kazis L, Ryan C (2020). Impact of work-related burn Injury on Social Reintegration outcomes: a life impact burn recovery evaluation (LIBRE) study. Arch Phys Med Rehabil.

[10] Nurczyk K, Chrisco L, Di Corpo M, Nizamani R, Sljivic S, Calvert C, Jones S, Cairns B, Williams F (2020). Work-related burn injuries in a Tertiary Care burn Center, 2013 to 2018. J burn care Research: Official Publication Am Burn Association.

[11] Jin Y, Ye P, Deng X, Yang L, Wang Y, Er Y, Wang W, Gao X, Ji C, Duan L. [Burn-related burden among Chinese population from 1990 to 2013]. Zhonghua Liu Xing Bing Xue Za Zhi = Zhonghua liuxingbingxue zazhi 2017, 38(6):767–71. 10.3760/cma.j.issn.0254-6450.2017.06.016 28647980

[12] Yang J, Tian G, Liu J, Bai H, Yang S, Ran M, Li H, Ma K, Yang S, Fu X (2022). Epidemiology and clinical characteristics of burns in mainland China from 2009 to 2018. Burns Trauma.

[13] Mohammadi A, Pakyari M, Seyed Jafari S, Tavakkolian A, Tolide-Ie H, Moradi Z, Kherad M (2015). Effect of burn sites (upper and lower body parts) and gender on extensive burns’ mortality. Iran J Med Sci.

[14] Dhopte A, Bamal R, Tiwari V (2017). A prospective analysis of risk factors for pediatric burn mortality at a tertiary burn center in North India. Burns Trauma.

[15] Chen L, He X, Xian J, Liao J, Chen X, Luo Y, Wang Z, Li N (2021). Development of a framework for managing severe burns through a 17-year retrospective analysis of burn epidemiology and outcomes. Sci Rep.

[16] Zhang Y, Zhang J, Jiang X, Ni L, Ye C, Han C, Sharma K, Wang X (2016). Hydrofluoric acid burns in the western Zhejiang Province of China: a 10-year epidemiological study. J Occup Med Toxicol (London England).

[17] Wang Y, Yu X, Qian W, Zhou D, Yang T, Wang S, He W, Luo G (2018). Epidemiologic Investigation of Chemical Burns in Southwestern China from 2005 to 2016. J burn care Research: Official Publication Am Burn Association.

[18] Han D, Wei Y, Li Y, Zha X, Li R, Xia C, Li Y, Yang H, Xie J, Tian S (2022). Epidemiological and clinical characteristics of 5,569 Pediatric Burns in Central China from 2013 to 2019. Front Public Health.

[19] Pompermaier L, Elmasry M, Abdelrahman I, Fredrikson M, Sjöberg F, Steinvall I (2018). Are there any differences in the provided burn care between men and women? A retrospective study. Burns Trauma.

[20] Karimi K, Faraklas I, Lewis G, Ha D, Walker B, Zhai Y, Graves G, Dissanaike S (2017). Increased mortality in women: sex differences in burn outcomes. Burns Trauma.

[21] Shi S, Yang H, Hui Y, Zhou X, Wang T, Luo Y, Xiang H, Shi X (2016). Epidemiologic characteristics, knowledge and risk factors of unintentional burns in rural children in Zunyi, Southwest China. Sci Rep.

[22] Chiwaridzo M, Zinyando V, Dambi J, Kaseke F, Munambah N, Mudawarima T (2016). Perspectives of caregivers towards physiotherapy treatment for children with burns in Harare, Zimbabwe: a cross-sectional study. Burns Trauma.

[23] Emond A, Sheahan C, Mytton J, Hollén L (2017). Developmental and behavioural associations of burns and scalds in children: a prospective population-based study. Arch Dis Child.

[24] Moolji J, Gill I, Varughese R, Adam B, Halloran K, Weinkauf J, Lien D, Mullen J, Hirji A. Successful long-term outcome after transplantation of lungs affected by smoke inhalation injury. The Annals of thoracic surgery 2021. 10.1016/j.athoracsur.2021.09.045 34699753

[25] Klifto K, Quiroga L, Hultman C (2019). Substance use and inhalation injury in adult burn patients: retrospective study of the impact on outcomes. Burns Trauma.

[26] Jähne M (2000). [25 years Cardona keratoprosthesis after severe chemical eye burns–long-term outcome of 4 eyes]. Klin Monatsbl Augenheilkd.

[27] Wang T, Nie C, Zhang H, Zeng X, Yu H, Shi S, Wei Z, Shi X (2018). Epidemiological Characteristics and Disease Burden of Burns in Children in Northern Guizhou, China. Chin Med J.

[28] Mo Y, Li X, Wang J, Chen C, He W, Guan H, Luo G, Liang G. [Summary of the 16th Chinese Symposium on Burn Medicine and the 2021 Congress of Burn Medicine Branch of China International Exchange and Promotion Association for Medical and Healthcare and the 2021 International Summit Forum of Burns in Chongqing]. Zhonghua shao shang za zhi = Zhonghua shaoshang zazhi = Chinese journal of burns 2021, 37(6):596–600. 10.3760/cma.j.cn501120-20210603-00210 PMC11917282 34167287

[29] National Bureau of Statistics of. the People’s Republic of China. Available at: http://wwwstatsgovcn.

[30] Arshi S, Sadeghi-Bazargani H, Mohammadi R, Ekman R, Hudson D, Djafarzadeh H, Delavari A, Sezavar H (2006). Prevention oriented epidemiologic study of accidental burns in rural areas of Ardabil, Iran. Burns: J Int Soc Burn Injuries.

[31] Zhu L, Zhang Y, Liu L, Jiang J, Liu Y, Shi F, Yi D (2013). Hospitalized pediatric burns in North China: a 10-year epidemiologic review. Burns: J Int Soc Burn Injuries.

[32] Ritenour A, Blackbourne L, Kelly J, McLaughlin D, Pearse L, Holcomb J, Wade C (2010). Incidence of primary blast injury in US military overseas contingency operations: a retrospective study. Ann Surg.

